# Uninvited Guests: Traditional Insect Repellents in Estonia used Against the Clothes Moth *Tineola bisselliella,* Human Flea *Pulex irritons* and Bedbug *Cimex lectularius*

**DOI:** 10.1673/031.010.14110

**Published:** 2010-09-13

**Authors:** Renata Sõukand, Raivo Kalle, Ingvar Svanberg

**Affiliations:** ^1^ Tartu University, Institute of Philosophy and Semiotics, Department of Semiotics, Tiigi 78, Tartu, Estonia; ^2^ Estonian Literary Museum, Vanemuise 42, Tartu, Estonia; ^3^ Uppsala Centre for Russian and Eurasian Studies, Uppsala University, Box 514, 751 20 Uppsala, Sweden

**Keywords:** human-insect relations, ectoparasites, repellents, herbal landscape, biocultural domains, local knowledge, ethnobiology

## Abstract

Extensive folklore records from pre-modern Estonia give us an excellent opportunity to study a variety of local plant knowledge and plant use among the peasantry in various parts of the country. One important biocultural domain where plant knowledge has been crucial was in the various methods of combating different ectoparasites that cohabited and coexisted with humans and their domestic animals. Some of these methods were widely known (world-wide, Eurasia, Europe, Baltic Rim), while others were more local. Here we discuss ways of reducing clothes moths *Tineola bisselliella* (Hummel) (Lepidoptera: Tineidae), human fleas *Pulex irritons* L. (Siphonaptera: Pulicidae) and bedbugs *Cimex lectularius* L. (Hemiptera: Cimicidae) with the help of plants. Various taxa used as traditional repellents have been identified. The use of plants as repellents and their toxic principles are also discussed from a comparative perspective.

## Introduction

Ectoparasitic insect species cohabitating with humans and their domestic animals have always played an important role in cultural history (Hanström 1933). The biocultural domains that have developed in the interaction between various kinds of parasites and human societies are probably the most intimate ‘activity contexts’ that exist (Balée 1994). In the pre-modern North it was actually considered healthy to have fleas and lice (Rosander 1970). Individuals living in contemporary industrially developed and postindustrial countries prefer life without these “annoying”, “blood-sucking” or “wool-eating” insects, whose presence in the household are associated with poor sanitary conditions and untidiness of the inhabitants (Mathlein and Tunblad 1971; Frykman and Löfgren 1987). Once they were part of everyday life, and they still are in many parts of the world (Ashford and Crewe 1998).

There are several ways to get rid of ectoparasites in homes and stables and on human and animal hairs. Simple manual methods of varying effectiveness have been common. A widespread approach to reduce bedbugs in northern Europe, including Estonia, was the use of a wooden board with many small holes drilled into it. The board was placed in the bed. The bedbugs lodged in the holes of the board after sucking blood from the sleeping person and could therefore easily be killed by pouring them into the fire in the morning. This method is known since the mid-eighteenth century (Isberg 1978; Thorsell and Tunón 2001). Another widespread method was to pick the parasites by hand. Old peasant women in Estonia often killed lice and bedbugs before bed-time in the evening, a common pastime in the country- side farmhouses still in the early twentieth century (Russwurm 1855 pp. 220, 232–233; Danell 1951; cf. Rosander 1970). Also magical ways of forcing bedbugs away from the home existed (Russwurm 1855 pp. 233; Söderbäck 1940).

Human history also records the use of many plant taxa as repellents and insecticides (Guarrera 1999; Argüello van de Putte 2005; Moore and Debboun 2007). Until the discovery of synthetic chemical insecticides in the 1930s, these plants were important in fighting parasites. Now we know that the use of synthetic insecticides may lead to several problems such as toxicity in humans, cattle and pets including groundwater contamination, disruption of natural biological control, destruction of wildlife, and the parasite's development of resistance to pesticides (Marco et al. 1986; Van Der Hoek 1998; Isman 2006). Therefore, many researchers are interested in using botanical insecticides instead for protection against insects in stored products (Isman 2006; Khoshnoud et al. 2008) or as mosquito repellents (Müller et al. 2009; Carroll and Loye 2006). But the historical use of plant-based repellents for protection against domestic parasites has been underestimated to date. The subject has been of interest to scholars in ethnobiology and economic botany for a long time. Carl Linnaeus documented local knowledge about various plant repellents during his expeditions through Swedish provinces (Svanberg 2005).

The aim of this article is to discuss ecologically safe plant repellents and insecticides used by peasants in Old Estonia against clothes moths *Tineola bisselliella* (Hummel) (Lepidoptera: Tineidae), human fleas *Pulex irritans* L. (Siphonaptera: Pulicidae) and bedbugs *Cimex lectularius* L. (Hemiptera: Cimicidae). Comparative perspectives with other countries in Northern Europe are also provided, where data are available.

### Ethnographic Settings

The Republic of Estonia is in Northern Europe. It is bordered to the north by the Gulf of Finland, to the west by the Baltic Sea, to the south by Latvia, and to the east by the Russian Federation. The territory of Estonia covers 45,227 km^2^ and is influenced by a temperate seasonal climate. The Estonians are a Finno-Ugric people, with the Estonian language exhibiting many similarities to Finnish (Raun 2001).

At the end of the nineteenth century, present-day Estonia was a small part of the Russian Empire, including the province of Estonia and almost half of Livonia. The population of Estonia was 893,558 according to the Russian census of 1881. General literacy at that time was relatively good, but only a few native Estonians had the possibility of obtaining a higher education. The Estonian language was used mostly among the rural population. A Swedish-speaking minority lived along the north-western Estonian coastal areas and islands as fishermen, seal hunters and peasants. Other minorities, including Germans, Russians and Tatars, were mostly urban dwellers (Ränk 1976).

The sanitary situation of the country at this time is poorly documented, though only a few buildings of the rural population had wooden floors and the dry toilet was introduced only in the early 1920s (Ränk 1980). Although some advice on repellents was occasionally given in early books on housekeeping (e.g. Karu 1903), human fleas and bedbugs did not seem to be a real concern until the late 1930s, when new methods were proposed in several official medicinal literary sources (Kask 1935; Leius 1938).

Nevertheless, peasants knew well how to protect themselves against these uninvited guests. They used specific plants, and named some of them according to the parasites they were used to repel. Local populations in the pre-modern context often had an excellent familiarity with the biological environment. This understanding of what Lévi-Strauss calls ‘science of the concrete’ does not only include those organisms and contexts that reflect cultural, economic and medicinal needs, but also a deep and detailed knowledge of the landscape and its biota (Lévi-Strauss 1962). Plants were necessary for the well-being of the peasantry. Therefore, the Estonian peasants, like rural populations all over Europe, developed and lived in a herbal landscape with domestic, semi-domestic and wild taxa of great use for them (Sõukand and Kalle 2010a; cf. Svanberg 2006).

### Methodology

Although the terms “ethnobotany”, “ethnozoology”, “ethnobiology” and “ethnoecology” were not coined until 1895, 1899, 1935 and 1954 respectively, the history of the ethnobiological field begins in northern Europe long before these dates. Even though the research did not develop into a separate academic discipline, many European scholars within botany, ethnology, and human geography, as well as advanced amateurs, made important contributions to ethnobiology (Svanberg et al. 2010). For instance, in 1888, Estonian folklorist and linguist Jakob Hurt (1839–1907) launched his famous appeal to “active sons and daughters of Estonia” to collect local folklore, initiating a long-lasting tradition. Among other requests (to collect songs, myths, and beliefs), he named 54 folk plant names (with Latin equivalent supplied for some) and asked to be sent popular descriptions of their use. Since that time, almost 1.5 million handwritten pages of folklore have been collected and stored in the Folklore Archives of Estonian Literary Museum in Tartu (www.folklore.ee/era/leidmine/index.html), including a considerable amount of local plant knowledge and plant lore. This study uses materials digitized for the Historical Estonian Herbal Medical Database (Historistlik Eesti Rahvameditsiini Botaaniline Andmebaas) (Sõukand and Kalle 2008) that covers all human medical herbal lore collected from 1888 to 1994, and includes information on the use of plants in cosmetics and against parasites.

Texts describing protection from three domestic parasites were selected from the digitized material. No voucher specimens were available to confirm the proper identification in the cited works, so the texts were first analysed according to a plant's native name. The species were determined in four different ways:

1) The scientific name of the plant was already given by the respondent, which is quite rare in the early material. More often the species were detected by botanists or other persons experienced in botany (G. Vilbaste, M. Ostrov, etc.) according to the specimen sent along with the folklore text. All the specimens were later lost or separated from the folklore texts.
2) In cases where the native plant name could be attributed to one particular species, the native plant name database compiled by Estonian natural scientist Gustav Vilbaste (1993) was used, as well as other available sources.
3) Often, vernacular names given in texts could be attributed to several species. The description of the plant recorded in the text or other context (e.g. parish where the text was collected) was taken into account in determining the correct plant among several possible variants.
4) In cases where a commonly used plant was identified at the genus level, which comprizes several very common species not distinguished by folk taxonomy, only genus was identified.

The complete information for each plant species or genus is given in Table 1, including family, the full scientific name, the Estonian vernacular name, use cases against all three insects and plant parts used, and preparation. Table 2 shows the list of plants that were attributed vernacular names related to insects in Estonian (according to Vilbaste (1993) as well as in Finnish (according to Suhonen 1936).

### The parasites

We will here consider three parasites and how they were treated. The human flea *P. irritons* is a cosmopolitan species. It is thought to have originated in South America and its original host may have been the guinea pig or peccary (Buckland and Sadler 1989). The flea bites many species of mammals and birds, including humans, domestic dogs, cats, etc. Besides botanical repellents, Estonian peasants used overall cleaning and the sauna for getting rid of fleas.

Larvae of the clothes moth *T. bisselliella* are known to be a damaging pest for humans, causing severe economic loss. They derive nourishment mostly from wool clothing (which was one of most commonly used cloths for peasants at colder times of the year), and from many other sources as well (feathers, fur, hair, leather). Besides botanical means, ventilation and aeration of clothing or bleaching in the sun was widely used to avoid damage to woolen clothing.

**Table 1.**
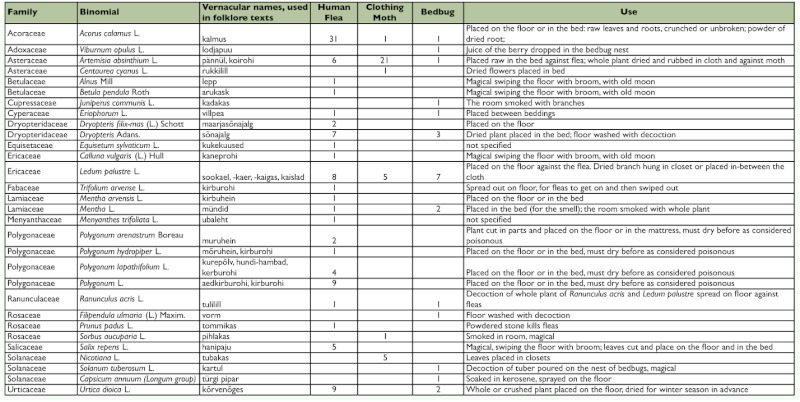
Repellent and insecticide plants used by native Estonians according to folklore data collected 1888–1994.

The bedbug *C. lectularius* lives by feeding on the blood of humans and other warm-blooded hosts. Its name comes from its preferred habitat around beds (walls, dark corners, etc.). Bedbugs are mainly active at night, sucking human blood. Their bites consist of a raised red bump or flat welt, and are often accompanied by very intense itching. Prior to the mid-twentieth century, bedbugs were quite common in Estonia, although botanical repellents were used in relatively few cases.

**Table 2.**
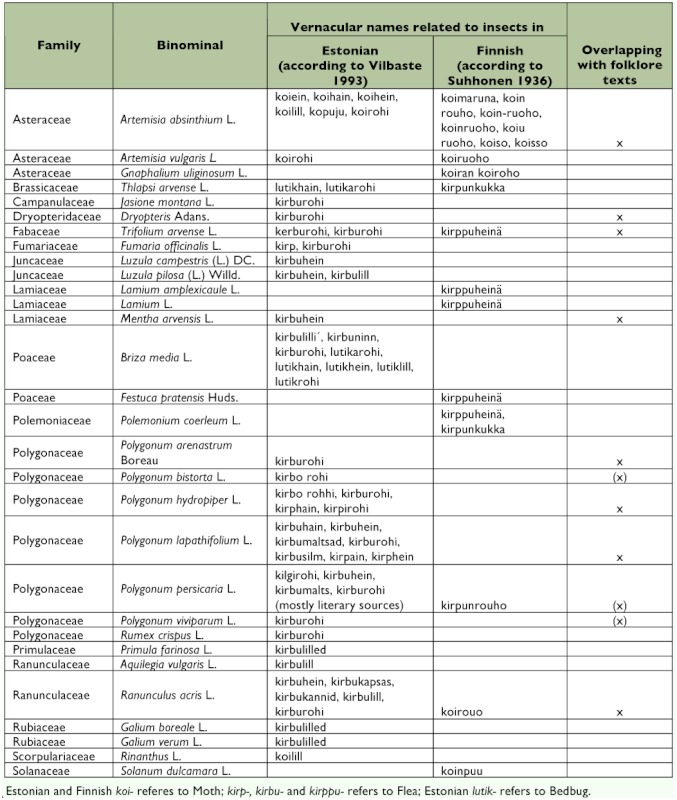
Estonian and Finnish names referring to Fleas, Moths and Bedbugs.

Peasants mostly used hot water and vapor as well as seasonal freezing to get rid of them (Isberg 1978).

## Results and Discussion

In this study 152 cases describing protection against these three domestic parasites were found. Of them, plant use against human fleas *P. irritons* was mentioned in 95 reports, the clothes moth *T. bisselliella* was mentioned in 34 reports, and the bedbug *C. lectularius* was mentioned in 23 reports.

As many repellent plants were suggested for all three or two of three parasites discussed, these plants and their indications are discussed in-depth. Plants having less than three indications and not bearing a name referring to an insect have been left out of the discussion as well as those whose insect-related name is attributed in less than three locations, as they could have been used as repellents in earlier times, but existing documentation of their names and uses cannot convincingly prove their use.

**Sweet flag** *Acorus calamus* L. (Acorales: Acoraceae). Originates in Southern Asia and Africa (Motley 1994; Lange 1999). It was introduced into the border region of Estonia probably as early as the 13th century (Lange 1999), and became well established in the wild. The plant has been valued medicinally since ancient times. In Estonian folk medicine *A. calamus* was used for treating a wide spectrum of diseases, including rheumatic disease, toothache, cough, and as an appetizer (Sõukand and Kalle 2008). The plant was used as a repellent mostly because of its aromatic properties. Its use was consistent over the entire researched period, and quite few literary sources propose its use. Many sources indicate that its effect is ‘too weak’ (Leius 1938). Raw or dried parts of sweet flag were placed on the floor or in the bed. Also, the rhizome was cut into pieces, dried and then ground into powder. It was used mostly against human fleas, but some texts also mention its use against clothes moth and bedbugs. The plant has no names related to ectoparasites. Most of its vernacular names refer to the Latin name (*kalmus, kalmujuur,* etc.).

**Figure 1.**
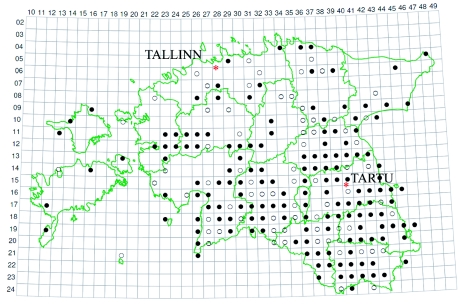
The distribution of *Acorus calamus* in Estonia, 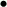 - The species has been found in 1971–2005, 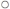 - Distribution data from 1921–1970 (Kukk and Kull 2005: 360). High quality figures are available online.

**Figure 2.**
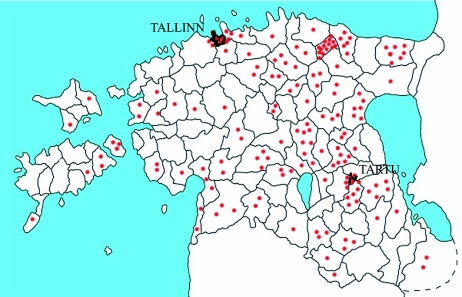
The distribution of all ethnomedical botanical knowledge related to *Accorus calamus,* according to HERBA. High quality figures are available online.

The Estonian populations of sweet flags are sterile triploids, which require direct human care for reproduction. *Acorus calamus* can be considered a consciously introduced plant in the Estonian herbal landscape as it has been cultivated and used for medicinal proposes. It grows in the southern and eastern parts of the country in areas with many small lakes. As can be seen in Figures 1 and 2, areas where sweet flag has been found are almost the same as the areas from which the folklore texts were collected. Exceptions are big towns, where the cloud of the dots is very dense; inhabitants of the town may have originated from rural areas where sweet flag was growing.

**Figure 3.**
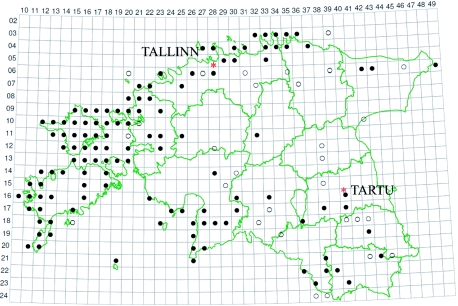
The distribution of *Artemisia absinthium* in Estonia, 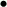 - The species has been found in 1971–2005, 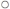 - Distribution data from 1921–1970 (Kukk and Kull 2005: 55). High quality figures are available online.

**Figure 4.**
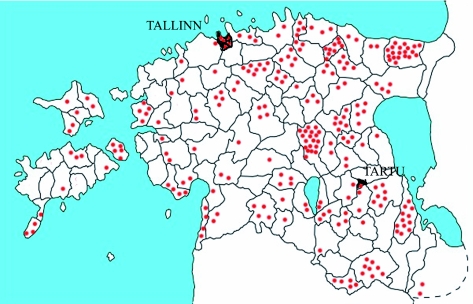
The distribution of all ethnomedical botanical knowledge related to *Artemisia absinthium* collected during the last century, according to HERBA. High quality figures are available online.

**Wormwood** *Artemisia absinthium* L. (Asterales: Asteraceae). Originates in temperate Eurasia and northern Africa. It has been known in northern Europe since medieval times and has acclimatized in Estonia and became native (Lange 1999). In Estonia wormwood grows along the coast and on the islands, while its distribution in other regions is diffuse. It grows in clumps and can survive in many conditions, although it seems to prefer bromes with human influence (wastes, grass verges etc). Though it has been observed only occasionally in wild areas of central Estonia (Figure 3), it is still widely used there (Figure 4). This highlights the fact that people cultivated wormwood in their garden for medicinal purposes.

As the plant has a characteristic scent, it was used to repel fleas and moths indoors all over the world. The whole plant was placed fresh in the bed against fleas. The whole plant was dried and then rubbed in cloth and fur against clothes moths, being the most important plant in protection against them in Estonia.

In addition to its use as a repellent, Wormwood was used in Estonian folk medicine as a universal medicinal plant applied to the treatment of various ailments such as cold, tuberculosis, rheumatic diseases, skin conditions and women's menstrual complaints as well as for stomach pains, upset stomach and as a digestive aid (Sõukand and Kalle 2008). It was also widely used for removing intestinal worms in all parts of Estonia. Provincial names given to Wormwood can be divided into two categories: names related to *koi* (“moth”) in the northern part of Estonia, and those related to the Russian name “*polõn*”, called *pännül* in southern and south-eastern Estonia (Vilbaste 1993). Its folk names also refer to the moth among the Swedish-speakers in Estonia and in Sweden, and among Finnish as well as German-speakers in many parts of Central Europe (Suhonen 1936; Marzell 1943; Danell 1951; Stenholm 1961; Svanberg 1998).

The related species, *Aretmisia vulgaris* L. was also named *koirohi,* “moth-herb” in Estonia and *koiruoho* in one parish in Finland (Vilbaste 1993; Suhonen 1936). Although this medicinal plant was used to treat several diseases, its usage as a repellent is not documented.

**Marsh rosemary** *Rhododendron tomentosum* Harmaja, Syn. *Ledum palustre* L. (Ericales: Ericaceae). This marsh plant is native to northern Europe, including Estonia. It grows in peaty soils and shrubby areas, and its distribution covers the entire country. In Estonian folk medicine it was mostly used against diseases whose origin were thought to be initiated by wet and cold conditions (coughs, tuberculosis, cold, rheumatic diseases, etc.), but its use was rather infrequent in comparison with other widely used medicinal plants (Sõukand and Kalle 2008).

However, as a repellent, it was fairly frequently used against all the insects mentioned in this paper, being the most popular agent against bedbugs. It was placed on the floor where it came in contact with the flea. Dried branches were also hung in the closet or placed in-between the bedclothes against clothes moths and bedbugs. Marsh rosemary has no folk names referring to insects; most of its names began with *soo*“swamp” or *raba-* “bog” (Raag 1984).

**Stinging nettle** *Urtica dioica* L. (Rosales: Urticaceae). This is a native taxon to Eurasia and North Africa, introduced into Estonia so long ago that is considered native. It is a widespread anthropochorous taxon which grows in clumps and prefers rich soils. It is found in the wild as well as in bromes with human influence. In Estonian folk medicine stinging nettle is widely used for treating rheumatic diseases, skin conditions, paralysis and cold (Sõukand and Kalle 2008). The stinging nettle has local names that overlap with the very similar Small nettle, *Urtica urens* L. However, neither of these names refers to insects (Vilbaste 1993), and the latter taxon was not utilized as an insect repellent. For use against fleas and bedbugs the whole or crushed plant was placed on the floor to repel the insects. Also, the whole plant was dried for use during the winter season.

**Knotweed** *Polygonum* L. (Caryophyllales: Polygonaceae). *Polygonum* is the only plant genus in which almost all species in Estonia have at least one provincial name related to the human flea. That may also be a reason why this plant family still bears the name *kirburohi,* “flea herb” in Estonian scientific nomenclature. Some of these species are quite difficult to differentiate, so for peasants they were all the same (Vilbaste 1993). Of them, *Polygonum amphibium* L., *Polygonum arenastrum* Boreau, *Polygonum hydropiper* L., *Polygonum lapathifolium* L., and *Polygonum persicaria* L. are country-wide apophytes (indigenous plants preferring environments affected by moderate to strong human impact). The species of the knotweed family were used exclusively against human fleas. The whole plant was cut, dried and then placed on the floor or in the bed. The raw plant was considered toxic, except for *P. arenastrum.* In other domains of folk medicine, the most common species of knotweeds were very rarely used, except again for *P. arenastrum,* which has become very popular since the 1970s (Sõukand and Kalle 2008).

Comparative data about their usage as repellents is lacking. In Norway, *P. persicaria* was locally called *loppegras* “flea-grass” because the seeds looked like fleas (Høeg 1974).

**Wood Ferns** *Dryopteris* Adanson (Dryopteridales: Dryopteridaceae). This genus includes about 250 species of ferns distributed in the temperate Northern Hemisphere, with the highest species diversity in eastern Asia. In Estonia, five species of wood ferns are present and four of them are widespread: *D.* *carthusiana* (Vill.) Fuchs, *D. cristata* (L.) Gray, *D. expansa* (C. Presl) Fraser-Jenk. & Jermy and *D. filix-mas* (L.) Schott. Estonians seldom differentiate species of *Dryopteris* which are not given insect-related vernacular names (Vilbaste 1993).

Although male-fern *D. filix-mas* was recommended as a vermifuge by all the Classic authors, its main use in Estonian folk medicine was against rheumatic diseases. However, many folklore texts mention its deworming effect as well (Sõukand and Kalle 2008). It was also employed as a repellent for human fleas (its dominate use) and for bedbugs. The leaves of the fern were placed on the floor or in the bed. Sometimes the floor was washed with a decoction of the whole plant. Probably this fern was attributed a repellent activity due to the sporophytes resembling small insects, but the repellent activity might have also been discovered during its use against rheumatic diseases, where the plant was placed in the bed.

**Tobacco** *Nicotiana* ssp. L. (Solanales: Solanaceae). This genus includes several taxa that are indigenous to North and South America. Brought to Estonia by itinerant peddlers in the seventeenth century, its cultivation started quite soon after that. The most popular cultivated species were *N. rustica* and *N. tabacum.* Although home cultivation of tobacco was prohibited in the 1920s, it has still been grown in some home-gardens in small quantities (Pallo 2002). No vernacular names for tobacco have been recorded by folklorists or linguists and no specimen vouchers exist, so it is difficult to decide the exact taxon that is being referred to in the archived folklore. In Estonian folk medicine tobacco was used mostly for repelling snakes or applied on snakebites and it was believed that the plant had supernatural power. Its smoke was also used against toothache and the leaves were placed on spots affected by skin diseases or scabies (Sõukand and Kalle 2008).

There are only five reports in the folklore records about tobacco leaves placed in closets among clothes to repel the clothes moth. The plant was probably primarily used because of the intense smell of the leaves and the mythic associations of the plant.

**Creeping willow** *Salix repens* L. (Malpighiales: Salicaceae). A native bush, distributed all over Estonia. It was used as a repellent for the human flea in magical rituals of sweeping the floor (*Alnus, Betula,* and *Calluna vulgaris* were used in a similar way*).* Leaves of creeping willow were also placed in between bedclothes. Although the bark of the representatives of the *Salix* family contains salicylic acid, the precursor to the non-steroidal anti-inflammatory drug aspirin, it was used only sparsely in Estonia for anti-inflammatory purposes (Sõukand and Kalle 2008). Creeping willow has no names referring to insects.

There was no comparative data available that indicates willow has been used for cleaning out fleas.

**Meadow buttercup** *Ranunculus acris* L. (Ranunculales: Ranuculaceae). This is one of the most common *Ranunculus* species across Europe and temperate Eurasia. It is regarded as native to Estonia and is found on both disturbed ground near habitation and in natural vegetation. The pollen of Buttercup may cause contact dermatitis, especially when it gets between cloth and human skin. This effect resembles the bite of a flea. Although the Historical Estonian Herbal Medical Database (Sõukand and Kalle 2008) contains only one text (from 1903) indicating the use of a decoction of the whole plants of *R. acris* and *R. tomentosum* spread on the floor against fleas, *R. acris* could have been used against fleas in earlier times, as it has many provincial names related to fleas (*kirbuhein, kirbukapsas, kirburohi* etc) (Vilbaste 1993).

**Mint** *Mentha* ssp. — Various taxa and hybrids of *Mentha* are distributed across Europe, Africa, Asia, Australia, and North America. They favor areas affected by human activity and many taxa are widely cultivated in home gardens. In Estonia, several mint hybrids occur and peasants did not usually differentiate between various taxa in their folk taxonomy. The whole plant was placed in the bed (for the scent), or the whole plant was burned and the smoke filled the room. Burning of plants is a common magical ritual and the branches of *Juniperus communis* and *Sorbus aucuparia* (both traditional holy trees in Estonia) were used for the same purpose.

**White clover** *Trifolium arvense* L. (Fabales: Fabaceae). This species of clover is native to all of Europe, except for the arctic zone, and to Western Asia. It grows in dry sandy soils and is typically found at the edge of fields, in wastelands or along roads. Although in the Historical Estonian Herbal Medical Database it is mentioned only once, it has some vernacular names related to fleas in several regions of Estonia and also in Finland. Its use is distinctly different from the customary usage of repellent plants. The plant was spread out on the floor in order to encourage the fleas to hide there, and then they were cleared away.

There was no comparative data available, but other plant species have been used for the same purpose in neighboring countries.

**Quaking-grass** *Briza media* L. (Poales: Poaceae). This is the only plant that has several vernacular names referring to fleas and bedbugs, but has no records of use against them. There can be two reasons for that. First, the plant was never actually used for this purpose and its names were attributed due to the similarity of the bedbugs to the quakinggrass spikelet. Another possibility is that the plant was actually used against parasites, but no one documented that use. With the decrease of magical plant rituals since the beginning of the nineteenth century some of magical uses may have been lost.

There was no comparative data from other parts of Europe indicating that this grass, popular among peasants in northern Europe for ornamental purposes, has been used as a repellent.

### Comparative perspectives

Five plants out of the twelve discussed have a specific scent that might be an important criterion for the selection for use against parasites. Only the first five taxa were mentioned by more than 10 respondents. The favorite plant for use against fleas was the naturalized sweet flag, *A. calamus,* leaving far behind the various native species of *Polygonum* that had many names related to insects. On the other hand, another naturalized alien, wormwood (*A. absintium*), was used the most against clothes moths, though it had insect-related names in Estonia as well as in Finland. The only plant that was used almost equally against all three insects was Marsh rosemary, *R. tomentosum.* It is notable, that of the twelve taxa discussed, this is the only species growing in the wetlands, where humans rarely lived. When such an effort is made to bring a plant home, it must be used for as many purposes as possible. That supports the theory of the herbal landscape established by humans in their near surroundings (Sõukand and Kalle 2010b).

Several plants described above have been used for the same purpose among the peasantry in the neighboring countries, Finland, Sweden, Denmark and Norway. Some of them have been known for a very long time, and their use has a wide distribution, in some cases almost worldwide.

Sweet flag *A. calamus* is an introduced plant in northern Europe. It has been known in the region probably since medieval times and has been recommended for various human and veterinary medicinal purposes in northern Europe since the 16^th^ century (Lange 1999; Svanberg 2006; Høeg 1974) and Danish sources mention its use against fleas among peasants in the nineteenth century (Brøndegaard 1978). It was known as a repellent already in ancient Indian sources as well. The oil made of its rhizomes has been employed until recently in India and various parts of Europe as an insecticide and repellent (Moore and Debboun 2007). South Asian sources also mention its use to repel fleas and protect clothes and grain from moths. More recent research shows that its dried rhizomes have active insecticidal properties (Motley 1994).

Wormwood, *A. absinthum* has been used as a dewormer, repellent and medicinal plant in many parts of the world (cf. Allen and Hatfield 2004; Guarrera 1999; Hayat et al. 2009). It was widely grown in pre-modern peasant villages and gardens in Scandinavia, and the spectrum of its use as a folk medicine and ethnoveterinary medicine was very wide (Svanberg 2006). Its use as repellent against clothes moths is well-known, not only through its vernacular names, but also in many written records. The oldest record of its use in Scandinavia is from Henrich Harpenstreng's herbal from around 1300, but there are many later sources that mention its actual use against moths among peasants in Denmark, Norway and the Åland archipelago (Brøndegaard 1980; Lange 1999; Høeg 1974; Liro 1915). Modern analysis shows that it is insecticidal and contains many repellent substances, for example thujone, terpenen 4-ol, linalool, nerol, geraniol, a-pinene, and 1.8-cineole (Moore et al. 2007; Kováts et al. 2010).

*Rhododendron tomentosum* is well-known as a repellent in northern Europe. Carl Linnaeus observed in 1742 how it was used among peasants in Södermanland against lice, and later also in Öland how it was “used in the pigsties when the swine have many lice” (Linnaeus 1745). Its use against lice has also been recorded from other parts of Sweden, Norway and Finland (Svanberg 1998; Alm and Iversen 2010). Marsh rosemary has been used for various folk medicinal purposes, and was actually integrated into school medicine by Linnaeus and his contemporaries (Linnaeus 1775). It contains myrcene and palustrol (Jaenson et al. 2005).

*Urtica dioica* has been placed in buildings against flies, especially among cattle in Sweden (Svanberg 1998). There are a few reports from Denmark that nettles were used as a repellent against various insects. Usually a freshly gathered bunch of nettles was hung up in the house (Brøndegaard 1979). The same is documented also from elsewhere in Europe (Vickery 1995; Allen and Hatfield 2004). Records from Norway say that the nettle plant was spread in beds in order to get rid of fleas (Høeg 1974). In Portugal it is still used as an insect repellent (Argüello van de Putte 2005). The insecticide activity of *Urtica dioica* is due to nicotine (Guarrera 1999).

*Ranunculus acris* is a toxic plant sometimes used as a folk medicine in northern Europe (Allen and Hatfield 2004). From Åland there is a record that it was seen as useful against bedbugs (Liro 1915).

Tobacco, *Nicotiana* ssp., has been widely used against lice in Sweden (Svanberg 1998: 308). It contains nicotine, a highly toxic alkaloid that has been used as an insecticide worldwide (Isman 2006).

Several species of mint were available in European pharmacies for various medicinal purposes already by the 18^th^ century. *Mentha* ssp. was, according to Linnaeus, put in small bags to keep fleas away from the bed (Linnaeus 1767). *Mentha arvensis* has been used in northern Sweden against lice (Svanberg 1998). There are records from Denmark that mint was placed in beds, cradles and chests against clothes moths and flies (the oldest record from the 15^th^ Century) (Brøndegaard 1980). From Great Britain there are folklore records that *M. spicata* was used to keep flies away from the house (Vickery 1995). The same plant was used in Norway against moths (Høeg 1974). In Portugal, *M. suaveolens* is still used by shepherds to drive away mosquitoes (Argüello van de Putte 2005).

Other taxa mentioned in the Estonian folklore records are not known as repellents or insecticides in other parts of Northern Europe.

### Conclusion

Studies of local botanical and ecological knowledge in Europe often reveal a pattern showing that the peasantry is influenced by “great traditions” with a long duration (cf. Heinrich et al. 2006; Svanberg 2007). Some of the plants used as repellents by Estonian country folk were already being utilized for the same purpose in ancient times. This knowledge spread, along with the plants, all over Europe. Some uses existed already in pre-historic times (*A. absinthium*); some probably developed during Medieval times or later (*A. calamus*; *Nicotiana* ssp.).

For the plants that have no comparative records of use, two different conclusions can be drawn. First, they were used only because of a name attributed according to some other characteristics of the plant (as *Polygonum* and *R. acris*) or only named, but not really used (as *B. media*). On the other hand, they could have been in use earlier and acquired the name from their actual use against fleas. Later on the less effective, more-lightly scented local taxa were replaced when more effective taxa were introduced. The fact that plants from the *Polygonum* family were relatively often in use, and this usage is documented by Vilbaste (1993), supports the second conclusion. The case of *T. arvense* is interesting because of its unusual use in repelling insects.
